# Laparoscopic liver resection: 5-year experience at a single center

**DOI:** 10.1007/s00464-013-3259-y

**Published:** 2013-11-07

**Authors:** Tran Cong Duy Long, Nguyen Hoang Bac, Nguyen Duc Thuan, Le Tien Dat, Dang Quoc Viet, Le Chau Hoang Quoc Chuong

**Affiliations:** Department of General Surgery, University Medical Center at Ho Chi Minh City, Ho Chi Minh City, Vietnam

**Keywords:** Hepatocellular carcinoma, Laparoscopic hepatectomy, Laparoscopic liver resection

## Abstract

**Background:**

Hepatocellular carcinoma (HCC) is a common cancer, especially in the Association of Southeast Asian Nations (ASEAN) region, where the prevalence of hepatitis virus infection is high. Liver resection is a potentially curative and popular therapy for HCC. Laparoscopic surgery using minimally invasive techniques potentially brings benefits to patients who need liver resection for HCC. This study aimed to evaluate the effectiveness, safety, and benefits of laparoscopic liver resection for HCC with long-term follow-up evaluation.

**Methods:**

This cohort study with 5-year results of total laparoscopic hepatectomy for HCC was conducted in one center. Patients with HCC were selected for laparoscopic liver resection by the same team. The operation also was performed by one team of surgeons. The follow-up protocol was similar to that for open surgery. The patients were scheduled to return for examination every 2 months after the operation. The data for the patients were collected and analyzed using SPSS software.

**Results:**

From January 2008 to December 2012, 173 enrolled patients with HCC underwent laparoscopic liver resection. The male-to-female ratio was 3:1. The mean age of the patients was 56 years (range 16–83 years). The follow-up period for 130 patients was 21.6 ± 16.0 months (range 0–60 months). The mean tumor size was 3.73 cm (range 2–10 cm). The stages of HCC according to the Barcelona Clinic Liver Cancer (BCLC) categorization were as follows: 0 (6 %), A1 (59.5 %), A2 (6.9 %), A4 (2.9 %), and B (27.2 %). Four patients required conversion to other techniques (2.3 %) because of the potential for major bleeding and tumor perforation. The types of resection were resection of one segment (segments 2, 3, 4, 5, 6, 7, and 8; 43.8 %), resection of two segments (posterior sector, anterior sector, segments 5 and 6, and left lateral sector; 47.9 %), resection of three segments (left and central liver; 4.7 %), and four segments (right liver; 3.6 %). The mean operation time was 112 ± 56 min (range 30–345 min), and the median blood loss was 100 ml (range 20–1,200 ml). The mean hospital stay was 6.5 ± 2.0 days (range, 3**–**19 days). No perioperative mortality occurred. The overall survival rates were 94.2 % at 1 year, 87 % at 2 years, 72.9 % at 3 years, 72.9 % at 4 years, and 72.9 % at 5 years. The mean overall survival time was 49.7 ± 2.1 months (range 45.5–53.9 months). The disease-free survival rates were 79.1 % at 1 year, 60 % at 2 years, 57 % at 3 years, 52 % at 4 years, and 26.3 % at 5 years. The mean disease-free survival time was 38.9 ± 2.6 months (range 33.9–44.0 months).

**Conclusion:**

Laparoscopic liver resection for HCC is feasible, safe, and effective, with good oncologic results. Major and anatomic hepatectomy are possible with improved skill and experience. Laparoscopic liver resection is a promising treatment option with minimally invasive benefits for HCC patients.

Hepatocellular carcinoma (HCC) is a common cancer, especially in the ASEAN region, where the prevalence of hepatitis virus infection is high. Liver resection is a potentially curative and popularized therapy for HCC. However, due to the special anatomic position of the liver, hepatectomy usually requires a very long incision, resulting in postoperative pain and discomfort. Laparoscopic surgery with minimally invasive techniques potentially brings benefits to patients who need liver resection for HCC.

Although laparoscopic liver resections have been performed for several years, the technique has not been widely used as expected. This type of operation has the following unsolved difficulties: surgical techniques that are not standardized, dissection and control of the hepatic hilus that still are challenging for laparoscopic surgeons, risk of massive bleeding and difficulty with bleeding control during liver parenchyma transection, establishment of oncologic principles in laparoscopic surgery for HCC, and long-term oncologic follow-up evaluation of the technique.

Resolving the aforementioned issues requires a study with a large number of patients and long-term follow-up assessment to evaluate the role of laparoscopic liver resection for HCC. This study aimed to evaluate the effectiveness, safety, and benefits of laparoscopic liver resection for HCC with long-term results.

## Materials and methods

This cohort study with 5-year results of total laparoscopic hepatectomy for HCC was conducted in one center. The operation was performed by one team of surgeons. The follow-up protocol was similar to that for open surgery. The patients returned for follow-up evaluation every 2 months after the operation. Patient data were collected and analyzed using SPSS software (SPSS, Chicago, IL, USA).

### Patient selection for laparoscopic liver resection

Tumors were free of major vessels, located in accessible segments of the liver, and amenable to curative resection. The tumors were smaller than 10 cm in the left liver and smaller than 5 cm in the right liver. Liver function was according to Barcelona Clinic Liver Cancer (BCLC) criteria and did not exceed class B. Patients whose overall status was categorized as American Society of Anesthesiology classes 1 to 3 were selected for laparoscopic liver resection.

### Operative technique

We performed pure laparoscopic liver resection.

### Patient position

For left lateral segmentectomy or anterior segmentectomy, the patient was placed in supine position with open legs. A posterior segmentectomy was performed with the patient in left lateral recumbent position.

### Trocar placement

We used five trocars and 45° oblique scopes. An infraumbilical trocar was used for the scope, and the camera holder stood between the patient’s legs. The two right-sided trocars were for the surgeon and the two left-sided trocars for the assistant. The port positions were dependent on the tumor location.

After mastering the learning curve, we improved and standardized our surgical technique. Extra-Glissonean dissection and anatomic liver resection were applied laparoscopically. We controlled the correlative Glissonean pedicle before transecting the liver parenchyma.

### Left lateral sectorectomy

The left hepatic pedicle was temporarily controlled by a vessel clamp (laparoscopic bulldog). The liver parenchyma was transected with a Harmonic scalpel. The Glissonean pedicles of segments 2 and 3 and the left hepatic vein were divided by a vascular stapler or a Hemlock clip. Finally, the vessel clamp at the left hepatic pedicle was released, and hemostasis in the transection plane was performed with bipolary cautery.

### Left or right hepatectomy (Fig. 1)

After cholecystectomy, we continued with extra-Glissonean dissection to expose the left or right pedicle. Then the hemi-hepatic pedicle was controlled temporarily with a vessel clamp (laparoscopic bulldog) to identify the discoloration on the surface of the liver. It is mandatory to ensure the anatomic border of the remnant liver and the intactness of the major vessels.

**Fig. 1:**
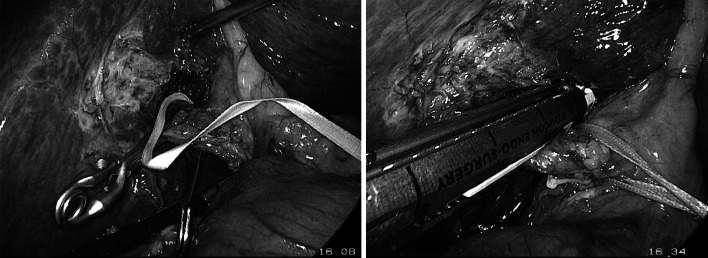
Laparoscopic right Glissonean pedicle transection after dissection

The Glissonean pedicle was divided by the vascular stapler. The liver parenchyma was transected with the Harmonic scalpel from inferior to superior and from anterior to posterior. Hemostasis on the liver plane was performed using bipolar cautery. The left or right hepatic vein was transected with the vascular stapler or controlled with the Hemlock clip. The specimen was withdrawn through the expanded infraumbilical trocar.

### Anatomic sectorectomy or segmentectomy in the right liver (Fig. 2)

We performed extra-Glissonean dissection to control the inflow to the correlative segment (anterior or posterior pedicle). The Glissonean pedicle was temporarily clamped with a laparoscopic vessel clamp. The borders of the segment then were identified by discoloration on the surface of the liver. The parenchyma was transected with the Harmonic scalpel. Bleeding points were controlled with bipolar cautery.

**Fig. 2:**
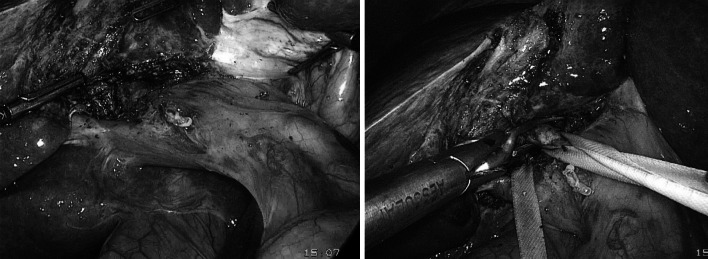
Laparoscopic Glissonean pedicle dissection

## Results

From January 2008 to December 2012, 173 patients with HCC were enrolled for totally laparoscopic liver resections. The male-to-female ratio was 3:1, and the mean of age of the patients was 56 years (range 16–83 years).

For all the patients, HCC was diagnosed according to the American Association for the Study of Liver Diseases (AASLD) criteria. The number of operations increased annually, including laparoscopic major liver resection. The numbers of patients undergoing totally laparoscopic liver resection from 2008 to 2012 were 21 in 2008, 36 in 2009, 39 in 2010, 31 in 2011, and 46 in 2012.

Totally laparoscopic liver resections for HCC were performed for 169 patients (97.7 %). The conversion rate was 2.3 %. Two patients underwent conversion to an open procedure due to high risk of major bleeding, and two patients underwent conversion due to high risk of tumor perforation. Two of these four patients had tumors located on segment 5 that caused major bleeding from the right portal vein. The remaining two patients also had tumors located on segment 5 that were encountered during the parenchymal transaction, which was converted to ensure a sufficient oncologic margin.

The mean tumor size was 3.73 cm (range 2–10 cm). According to the BCLC staging system, 3.5 % of our patients had very-early-stage disease (0), 69.3 % had early-stage disease (A), and 27.2 % had intermediate-stage disease (B) involving a single tumor larger than 5 cm in diameter (Table 1).

**Table 1:** Clinicopathologic patient data

Variables	Frequency	Range/percentage
Mean age (years)	56.54	16–83
Sex (male/female)		
Mean tumor size (cm)	3.73	2–10
Stage Followed BCLC (%)		
0	6	3.5
A1	103	59.5
A2	12	6.9
A3	0	0
A4	5	2.9
B	47	27.2
Mean operation time (min)	112 ± 56	30–345
Median blood loss (ml)	100	20–1,200
Surgical margin		
Close	10	5.9
<1 cm	13	7.7
1–2 cm	89	52.7
>2 cm	57	33.7
Specimen pathologic differentiation		
Good	30	17.3
Moderate	79	45.7
Poor	64	37.0
Positive margin/negative margin	4/165	2.4/97.6
Hospital stay (days)	6.5 ± 2.0	3–19
Complications		
Ascites after surgery	1	0.6
Pneumonia	1	0.6
Bile leakage	2	1.2
Peri-operation mortality	0	0
Mean follow-up time (months)	21.6 ± 16.0	0–60
Follow-up lost patients	130	39
Mean disease-free survival (months)	38.9 ± 2.6	33.9–44.0

Table 2 shows the types of resection performed for our patients. The mean operation time was 112 ± 56 min (range 30–345 min). The median blood loss was 100 ml (range 20–1,200 ml), and the mean hospital stay was 6.5 ± 2.0 days (range 3–19 days) (Table 3).

**Table 2:** Type of resection

Type of resection	*n*	%
One segment		
Segment 2	8	4.7
Segment 3	6	3.6
Segment 4	10	5.9
Segment 5	14	8.3
Segment 6	27	16.0
Segment 7	7	4.1
Segment 8	2	1.2
Two segments		
Posterior sector	7	4.1
Anterior sector	2	1.2
Segments 5 & 6	14	8.3
Left lateral sector	58	34.3
Three segments		
Left liver	7	4.1
Central segments of liver	1	0.6
Four segments		
Right liver	6	3.6
Total	169	100

**Table 3:** Surgical factors

Variables	Frequency	% Range
Mean operation time (min)	112 ± 56	30–345
Median blood loss (ml)	100	20–1,200
Surgical margin		
Close	10	5.9
<1 cm	13	7.7
1–2 cm	89	52.7
>2 cm	57	33.7
Specimen pathologic differentiation		
Good	30	17.3
Moderate	79	45.7
Poor	64	37.0
Positive margin/negative margin	4/165	2.4/97.6
Hospital stay (days)	6.5 ± 2.0	3–19
Complications		
Ascites after surgery	1	0.6
Pneumonia	1	0.6
Bile leakage	2	1.2
Perioperation mortality	0	0

**Fig. 3:**
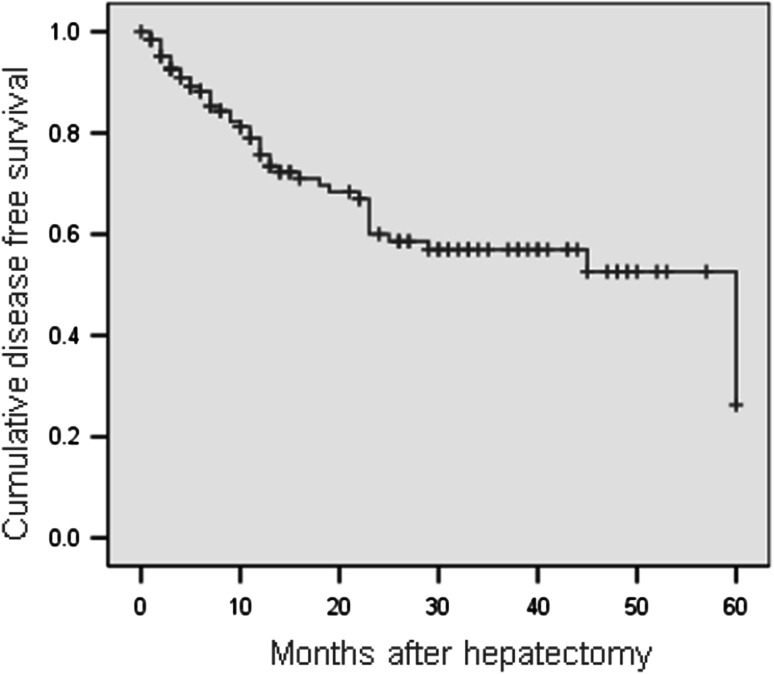
Disease-free survival curve (Kaplan–Meier)

**Fig. 4:**
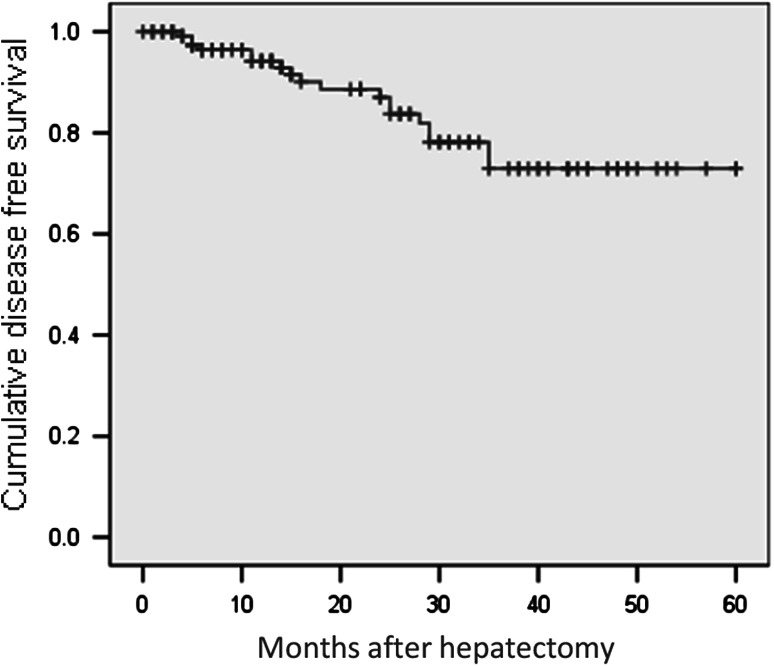
Overall survival curve (Kaplan–Meier)

During the follow-up period of 21.6 ± 16.0 months (range 0–60 months), we lost 39 patients. The disease-free survival rates in this study were 79.1 % at 1 year, 60 % at 2 years, 57 % at 3 years, 52 % at 4 years, and 26.3 % at 5 years (Fig. 1). The mean disease-free survival time was 38.9 ± 2.6 months (range 33.9–44.0 months). The overall survival rates were 94.2 % at 1 year, 87 % at 2 years, 72.9 % at 3 years, 72.9 % at 4 years, and 72.9 % at 5 years (Fig. 2). The mean overall survival time was 49.7 ± 2.1 months (range 45.5–53.9 months) (Figs. 3, 4).

## Discussion

In our center, we initiated our laparoscopic liver resection experience in 2005. The surgical techniques have been standardized and applied consistently since 2008. In this study, 173 consecutive patients with HCC who underwent totally laparoscopic liver resection were followed up for evaluation of the long-term results.

### Indication and feasibility of laparoscopic liver resection

Hepatocellular carcinoma was diagnosed according to the criteria of AASLD [1]. At the beginning, laparoscopic liver resection was indicated for peripheral tumor, especially for tumor located on the left lobe of the liver. For tumor in segment 2, 3, or 6, it is easier to apply the laparoscopic technique because liver mobilization is not required, the plane of transection is straightforward, and bleeding is controlled more easily. Laparoscopic liver resection helped patients to avoid the long incision needed in open surgery.

For tumor in the left lateral sector, we used the vascular stapler to divide the Glissonean pedicle and the left hepatic vein. This technique is feasible and safe, with short operative times. The current study showed that left lateral sector resection accounts for the largest percentage (34.5 %) of the operation. The World Consensus Conference on Laparoscopic Surgery for Liver Resection 2008 accepted laparoscopic left lateral sector resection as the standard operation for HCC [2].

Despite their peripheral position, tumors in segment 4b or 5 are in close proximity to adjacent major structures of the hepatic pedicle. In addition, the transection plan zigzags and is not a straight line and thus is more difficult.

In this study, we had seven tumors on segment 7 (4.1 %). We performed laparoscopic atypical liver resection successfully in all these patients. However, we recognize that this is quite difficult because it requires complete mobilization of the right liver and because the nonanatomic segment 7 resection is harder to perform.

For most of the patients, we were successful in completing a totally laparoscopic resection. Only in four patients (2.3 %) was this technique not possible for tumors located in segment 5. Two of these four patients underwent conversion due to difficulty controlling bleeding, and the remaining two underwent conversion because the surgical margin was too close to the portal vein. No emergency conversion to an open surgical technique was performed. Temporary clips or suture for hemostasis before a change to another technique was safe and suitable. Other techniques such as hand-assisted or hydrid technique were beneficial to the patients.

Based on our results, laparoscopic liver resection of HCC located on peripheral segments (segments 2, 3, 4a, 5, and 6) and not too close to major structures is safe and feasible, consistent with the literature [2–6].

Recently, we applied the intrahepatic Glissonean approach for dissection of the Glissonean pedicles to each segment separately before transecting the parenchyma. The approach was similar to the approach described by Machado et al. [3] and Cho et al. [4]. We considered this technique to be useful in laparoscopic surgery for help in identifying the anatomic borders of the segment with intent for resection, for selective control of the hepatic inflow, for reduction of ischemia to the remnant liver, for facilitation of the anatomic resection, and for help in ensuring the oncologic margin.

Several reports have shown the feasibility of major liver resections [5–10] and anterosuperior sectorectomy [11–13]. Most authors agree that laparoscopic surgery for major liver resection has complications similar to those of open surgery but offers the benefits of laparoscopic surgery [14] .

After increasing our experience with more straightforward laparoscopic liver resections, we broadened our indication for laparoscopic liver resections to major liver resection and anatomic liver resection. Our study had six patients (3.6 %) with right liver resection, seven patients (4.1 %) with left liver resection, seven patients (4.1 %) with posterior sectorectomy, two patients (1.2 %) with anterior sectorectomy, and one patient (0.6 %) with central hepatectomy.

The mean tumor size in our study was 3.73 cm (range 2–10 cm). Tumor size was not a critical factor in choosing between laparoscopic and open liver resection. However, we agree that large tumors impeded the operative techniques and increased the risk of tumor perforation. Tumor location and relation to other structures are more important in the choice of the operation.

Most cases of HCC have developed in a background of cirrhosis or chronic hepatitis. In our study, most of the patients had cirrhosis. However, several authors have recognized that laparoscopic liver resection has more benefits for patients with cirrhosis. Especially, postoperative ascites were less frequent because of the minimal invasion to lateral circulation [15] .

### Parenchymal transection and blood loss control

After selective control of the hepatic inflow, we transected the parenchyma with the Harmonic scalpel. Hemostasis was secured with bipolar cautery, and large vessels were clipped with Hemolock clips and divided. Application of this technique was effective, leading to short operative times and reducing the ischemia to the remnant liver. Our mean operative time was 112 ± 56 min (range 30–345 min).

Some authors have used the laparoscopic cavitron ultrasonic surgical aspirator (CUSA), a meticulous hemostatic instrument that helps to reduce bleeding but prolongs the operative time. In our study, the median blood loss was 194 ml (range 20–1200 ml). Selective control of the hepatic inflow with the intrahepatic Glissonean approach followed by parenchymal transection with the Harmonic scalpel according to the anatomic border reduces blood loss and facilitate laparoscopic surgery.

### Specimen pathologic result and surgical margin

Our goal for the surgical margin was a distance of at least 1 cm from the tumor. Based on the location of the tumor identified on imaging studies, we planned the appropriate liver resection with a margin greater than 1 cm in mind, ensuring adequate remnant liver volume. The surgical margins for 5.9 % of the tumors were too close to the tumor, and 7.7 % of the tumors had a margin of less than 1 cm. In these instances, the tumor was located too close to major vessels.

As Han et al. [16] reported, when intraoperative ultrasound is used to identify the tumor location and confirm the expected margin, sometimes this margin cannot be obtained with laparoscopic surgery. In laparoscopic surgery, tactile feedback is not possible for identification of the deep tumor and the tumor located too close to major vessels. Others have reported that 13 % of surgical margins are less than 1 cm from the tumor [17, 18]. These authors have concluded that this rate is similar to that in open surgery.

### Hospital stay

The hospital stay in our study was 6.5 ± 2.0 days (range 3–19 days). In other studies [19, 20], hospital stay in laparoscopic group was shorter than in the open group. For peripheral tumors, such as tumor in left lateral segments or segment 6, the hospital stays were 3 days. Moreover, the patients felt less pain postoperatively and returned to normal activities more quickly.

### Complications

No perioperative mortality occurred in our study. Complications occurred such as burden postoperative ascites (0.6 %), bile leak (1.2 %), and pneumonia (0.6 %). Nguyen et al. [9] reviewed 127 published papers on laparoscopic liver resection and found a cumulative mortality rate of 0.3 % and a morbidity rate of 10.5 %. The liver-specific complications included bile leaks (1.5 %), transient liver ascites (1 %), and abscesses (2 %). Our study had two cases with major bleeding intraoperatively that required a conversion of operative technique but no emergency conversion. Several reports described complications of laparoscopic liver resection, with most of them suggesting that open and laparoscopic surgeries do not differ significantly in terms of complications [21].

### Survival and recurrence

The recurrence and survival rates were the most important factors in the treatment of HCC with laparoscopic liver resection. Our follow-up protocol included reexamination of all patients every 2 months. Throughout the study, 130 patients were followed up. During the study period, 39 patients died. We had no peritoneal or port-site recurrence in the current study. The disease-free survival rates were 79.1 % at 1 year, 57 % at 3 years, and 26.3 % at 5 years. In most cases, recurrent HCC tumors were diagnosed early and treated by reoperation, Radio Frequency Ablation, or Transcatheter arterial chemoembolization to prolong survival.

The overall survival rates in this study were 94.2 % at 1 year, 72.9 % at 3 years, and 72.9 % at 5 years. From a European perspective, Kluger and Cherqui [22] reported the overall survival rates after liver resection for 163 patients with HCC to be 92.6 % at 1 year, 68.7 % at 3 years, and 64.9 % at 5 years and the disease-free survival rates to be 77.5 % at 1 year, 47.1 % at 3 years, and 32.2 % at 5 years.

In 2009, Nguyen et al. [9] in their review of laparoscopic liver resections reported 5-year overall survival rates after laparoscopic liver resection ranging from 50 to 70 % and disease-free survival rates ranging from 31 to 38.2 %. These results were comparable with those for open surgery. Sarpel et al. [20] and Ito et al. [19] conducted a retrospective case-matched study including comparable factors such as degree of cirrhosis and tumor characteristics. They suggested that no significant difference in outcome existed between the two groups.

## Conclusion

Laparoscopic liver resection for HCC is feasible, safe, and effective, with good oncologic results. Major and anatomic hepatectomy can be performed more common by improving skill and experience. Laparoscopic liver resection is a promising treatment option with mini-invasive benefits for HCC patients.
